# The CRISPR-Cas System and Clinical Applications of CRISPR-Based Gene Editing in Hematology with a Focus on Inherited Germline Predisposition to Hematologic Malignancies

**DOI:** 10.3390/genes15070863

**Published:** 2024-07-01

**Authors:** Rina Kansal

**Affiliations:** 1 Molecular Oncology and Genetics, Diagnostic Laboratories, Versiti Blood Center of Wisconsin, Milwaukee, WI 53233, USA; rinakansal@msn.com; 2 Department of Pathology and Anatomical Sciences, The University at Buffalo, Buffalo, NY 14260, USA

**Keywords:** CRISPR-Cas, gene editing, gene therapy, hematologic cancers, inherited germline predisposition to cancer, bone marrow failure, acute myeloid leukemia, myelodysplastic syndromes, myeloid neoplasms, DNA repair

## Abstract

Clustered regularly interspaced short palindromic repeats (CRISPR)-based gene editing has begun to transform the treatment landscape of genetic diseases. The history of the discovery of CRISPR/CRISPR-associated (Cas) proteins/single-guide RNA (sgRNA)-based gene editing since the first report of repetitive sequences of unknown significance in 1987 is fascinating, highly instructive, and inspiring for future advances in medicine. The recent approval of CRISPR-Cas9-based gene therapy to treat patients with severe sickle cell anemia and transfusion-dependent β-thalassemia has renewed hope for treating other hematologic diseases, including patients with a germline predisposition to hematologic malignancies, who would benefit greatly from the development of CRISPR-inspired gene therapies. The purpose of this paper is three-fold: first, a chronological description of the history of CRISPR-Cas9-sgRNA-based gene editing; second, a brief description of the current state of clinical research in hematologic diseases, including selected applications in treating hematologic diseases with CRISPR-based gene therapy, preceded by a brief description of the current tools being used in clinical genome editing; and third, a presentation of the current progress in gene therapies in inherited hematologic diseases and bone marrow failure syndromes, to hopefully stimulate efforts towards developing these therapies for patients with inherited bone marrow failure syndromes and other inherited conditions with a germline predisposition to hematologic malignancies.

## 1. Introduction

The treatment of genetic diseases has begun to be transformed since the Nobel Prize-winning discovery of the clustered regularly interspaced short palindromic repeats (CRISPR)/CRISPR-associated (Cas) protein 9/single-guide RNA (sgRNA)-based targeted gene-editing system in 2012 [1,2,3]. With >21,000 entries for “CRISPR editing” and close to 50,000 entries for “CRISPR” in PubMed literature at this time, clinical research is being actively pursued for CRISPR-based gene-editing therapies (reviewed in [4,5]). The CRISPR-Cas system is well-understood by microbiologists, biologists, and scientists who use it in their work. The history of its discovery is fascinating, highly instructive, and inspiring for students of science and medicine to apply the lessons learned throughout this discovery journey for future discoveries and innovations to benefit society. When used responsibly, this transformative technology, with its ongoing advances, combined with other significant discoveries and advances in medicine has the potential to cure many patients of diseases that are hard to cure or have evaded cure.

Many hematologic diseases, including non-malignant genetic diseases and malignancies, have the potential to be cured by a hematopoietic stem cell transplant (HSCT), a procedure that replaces the diseased bone marrow by infusing healthy long-term hematopoietic stem cells that engraft, proliferate, and differentiate to grow into healthy bone marrow and blood cells. An HSCT may be allogeneic or autologous, depending on the source of the donor hematopoietic stem cells. An allogeneic HSCT is effective but requires a matched donor, and only up to 30% of patients who need an allogeneic transplant have a matched donor in their family [6,7]. The lack of a matched donor leads to significant clinical challenges [8,9]. The chance of finding a matched donor outside of the family ranges from 29% to 79%, depending on the patient’s ethnic background [7,10]. In addition, an allogeneic HSCT carries the risk of immunological complications such as graft-versus-host disease, which can be fatal [8,9]. Graft-versus-host disease may be acute or chronic, depending on the clinical features [11]; the acute form of this disease showed 70% mortality in 2022 [12]. Chronic graft-versus-host disease was found to increase in incidence from 1995 to 2007 [13]; it remains the leading cause of late mortality other than relapsed disease after an allogeneic HSCT [14,15,16]. In contrast, an autologous HSCT uses the patient’s own stem cells, thus eliminating the risk of graft-versus-host disease. Autologous HSCT can be given to older individuals who may not be able to go through an allogeneic HSCT, but it is not as effective as an allogeneic HSCT [8]. Clinical research using autologous HSCT with edited autologous hematopoietic stem cells, i.e., gene therapy, has been performed in the last three decades in hematologic diseases with an underlying genetic cause (reviewed in [9]). The first CRISPR-Cas9 single-guide RNA (sgRNA)-based gene-editing therapy to treat patients with severe sickle cell disease was approved in the UK [17] in November 2023, and in the USA [18] and Europe [19] in December 2023. 

This unprecedented landmark accomplishment for science, technology, and medicine and continuous advances in gene-editing techniques and discoveries in medicine provide hope for effective therapies and continued applications for patients with other inherited hematologic diseases. Inherited bone marrow failure syndromes and germline predispositions to hematologic malignancies comprise a diverse and unique group of inherited conditions that are currently potentially curable only by an allogeneic HSCT, as recently reviewed [20,21]. For clarification, inherited or genetic hematologic diseases also include other diseases such as hemophilia that do not progress to any malignancy; this paper is focused on those inherited hematologic diseases that have the predisposition to progress to malignancy. These diseases are discussed in a subsequent section in this article and are grouped to include (1) inherited bone marrow failure diseases, which often affect the pediatric age group but may also be diagnosed in adults, including Fanconi anemia, Diamond–Blackfan anemia, Schwachman–Diamond syndrome, dyskeratosis congenita, and other telomere biology disorders, severe congenital neutropenia, and congenital amegakaryocytic thrombocytopenia, and more recently described diseases such as *ERCC6L2* inherited bone marrow failure; (2) genetic syndromes with a predisposition to hematologic cancer, including Li–Fraumeni syndrome, Lynch syndrome, constitutional mismatch repair deficiency syndrome, Bloom syndrome, Werner syndrome, ataxia telangiectasia, Nijmegen breakage syndrome, DNA ligase 4 deficiency, and the RASopathies; and (3) familial acute myeloid leukemia (AML) and myelodysplastic neoplasms, including AML with germline *CEBPA* mutations, familial platelet disorder with propensity to develop myeloid malignancies (*RUNX1*-related), *ANKRD26*-related thrombocytopenia, *ETV6*-related thrombocytopenia, AML or myelodysplastic neoplasms with *DDX41* mutations, and pediatric AML or myelodysplastic neoplasms with germline *GATA2*, *SAMD9*, and *SAMD9L* mutations (reviewed in [20,21]). 

Most germline predispositions described in this paper have the potential to transform into AML. AML is the most common disease for which allogeneic hematopoietic stem cell transplantation is performed, with the timing being during the first complete remission after treatment for AML [22]. The worldwide incidence of AML transplants more than doubled from 2006 to 2016 [22], with steadily increasing rates of allogeneic HSCT for patients with AML in North America and Europe [23]. Conceivably, many of these inherited states mentioned above with a germline predisposition and having the potential to progress to an aggressive hematologic malignancy such as AML would have the potential to be cured by an autologous HSCT with gene-edited hematopoietic stem cells, with an example published on 5 June 2024, for CRISPR-Cas9-mediated gene therapy for severe congenital neutropenia, an inherited bone marrow failure disease [24]. The purpose of this paper is three-fold: first, to briefly describe chronologically the discovery of the CRISPR-Cas system and CRISPR-Cas9 single-guide RNA (sgRNA)-based gene editing that led to the landmark approvals in 2023, with the reason explained above for including this history in a chronologic order; second, to briefly describe the current state of clinical research using CRISPR-based gene editing in hematologic diseases, including innovative preclinical advances for immunotherapies to treat aggressive hematologic malignancies such as AML and T-lymphoid cell neoplasms, preceded by a brief description of the base-editing and prime-editing tools being used in the clinical research described in this article; and third, to present the current progress in gene therapies in inherited hematologic diseases and bone marrow failure syndromes, to hopefully initiate further discussions in the scientific and expert medical communities toward applying gene-editing therapies, i.e., autologous HSCT with gene-edited hematopoietic stem cells, in individuals with a germline predisposition to hematologic malignancies.

## 2. The CRISPR-Cas System in Prokaryotes

This section and the next one describe a brief historical background of the discovery of the CRISPR-Cas immune defense system in prokaryotes and the development of CRISPR-Cas9-sgRNA-based gene-editing tools, followed by a summary of additional CRISPR-Cas systems and CRISPR genome-editing technologies developed by modifications of CRISPR-Cas that are being used in current clinical research. 

### 2.1. Discovery of the CRISPR-Cas System

The first CRISPR DNA sequences were discovered in *Escherichia coli* bacteria by traditional Sanger sequencing by Ishino and colleagues in Japan in 1987 [25] (p. 5432) and subsequently, in two species of Archaea by Mojica and colleagues in Spain in 1993 [26] and 1995 [27]. At that time, the significance of these newly discovered sequences was unknown. Archaea are single-celled microorganisms without a nucleus, similar to prokaryotes. Mojica, a Ph.D. student in the early 1990s, was studying *Haloferax mediterranei*, an Archaean species resistant to high-salt water. Upon sequencing the microorganism, he found the 30-base pair (bp) clustered repeats that intrigued him since they had not been described earlier in any microbes. These repetitive repeats included short, inverted palindromic repeats, as shown in a subsequent review article [28]. He searched the previous literature manually as was then the norm and found the Japanese paper published in 1987 that had described similar clustered repeats in a different microbe, a Gram-negative bacterium. The presence of these repeats in diverse organisms convinced him that these clustered DNA repeats were conserved and had to have an important function in unicellular microbes, whose entire genome size is not huge. He subsequently continued to study various microorganisms to decipher the significance of the clustered repeats [28,29]. The clustered regularly spaced palindromic repeats were initially called tandem repeats (TREPs) and short regularly spaced repeats (SRSRs). The name CRISPR was suggested by Mojica and accepted in 2002 [30] after Jansen and colleagues in the Netherlands found similar clustered repeats in Archaea and bacteria [27,28,29,30,31]. 

The first four CRISPR-associated (Cas) genes, *cas1*, *cas2*, *cas3*, and *cas4*, were discovered near the CRISPR sequences in 2002 [30]. Thus, the function of the encoded Cas proteins was suggested to be related to the CRISPR sequences by Jansen and colleagues [30]. Later, in 2005, Haft and colleagues identified 41 additional Cas protein families in the first 200 types of prokaryotes whose genomes had been sequenced by then, for a total of 45 Cas protein families, including the previously described four core Cas proteins, Cas1–Cas4 [32]. All Cas proteins were identified only in microbes that harbored CRISPR loci. These 45 protein families included (1) six core Cas proteins, Cas1, Cas2, Cas3, Cas4, Cas5, and Cas6, with the same names for the corresponding genes; (2) eight additional CRISPR-Cas subtypes with the family names specific for the microorganism containing only that CRISPR locus, including *E. coli* (with the genes *cse1*, *cse2*, *cse3*, *cse4*, and *cas5e*), *Yersinia pestis* (with the genes *csy1*, *csy2*, *csy3*, and *csy4*), and *Neisseria meningitidis* (with the genes *csn1* and *csn2*); and (3) one repair-associated mysterious protein (RAMP) superfamily [17]. Cas1 was the most often associated with CRISPR loci [32]. 

### 2.2. Discovery of CRISPR as a Mediator of Adaptive Immunity in Microbial Organisms

The 24–40 nucleotide clustered repetitive sequences were recognized by intervening non-repetitive regular spaces of sizes similar to the recurring sequences [33]. Archaea and prokaryotes are targets of viruses. That led to the eventual discovery of the biological function of these repeats in microbes. Mojica continued to study microbes and began to look at databases in the early 2000s to find matches of the repeats he had discovered with the sequences of bacteriophages. He eventually found a match between the intervening spacer sequences and a bacteriophage to which the microbe containing the spacer sequences was resistant. Then, Mojica and his colleagues studied about 4500 CRISPR spacer sequences from 67 strains of 36 prokaryotes to find multiple matches between the intervening spacer sequences and bacteriophages and plasmids. They found 88 spacer regions from 25 strains to be similar and to represent foreign DNA in the genomes of Gram-positive and Gram-negative bacteria and Archaea. In total, 47 of these spacer DNA segments corresponded to bacteriophages, 10 to plasmids, and the remaining 31 to chromosomal DNA not directly related to foreign DNA [33]. They tried to publish their findings in highly cited journals but were rejected and eventually published in February 2005 in a lesser-known journal [28,29,33]. 

One month later, a French group working on *Yersinia pestis* published similar findings in March 2005 [34]. Both the Spanish and French groups found the sequences of the intervening spacers in CRISPR loci to be homologous to sequences of bacteriophages, prophages, and plasmids [33,34]. They described that the CRISPR loci served as a self-defense mechanism in prokaryotes to prevent the entry of foreign genetic transmissible units [28,29,33,34]. 

In August 2005, another French group studying CRISPR spacers in the dairy bacterium *Streptococcus thermophilus* reported that the spacers contained extrachromosomal elements found in bacteriophages and plasmids [35]. They proposed that the CRISPR-associated *cas* genes fragmented foreign DNA as a preventive function and hypothesized that this function of the *cas* genes led to the formation of CRISPR sequences [35]. 

Finally, in 2007, Barrangou and colleagues provided experimental proof of the function of CRISPR and *cas* genes as an adaptive immunity mechanism in prokaryotes [36]. They studied *S. thermophilus* strains and showed that a viral challenge led the bacteria to integrate spacers derived from phage sequences into their genomes. Also, adding or deleting specific spacers led to phage-resistant or phage-sensitive properties, respectively [36]. Further, they showed that inactivating two different *cas* genes resulted in losing phage resistance in one gene, *cas5*, but did not alter that property in the other, *cas7*, gene, hypothesizing that *cas7* might be involved in creating new spacers [36]. In a subsequent study published in February 2008, they showed that new spacers were acquired in four strains in response to bacteriophages. Specific motifs associated with precursor spacers in *S. thermophilus* were also noted [37]. 

### 2.3. Mechanisms of CRISPR: *How* These Loci Work to Produce Adaptive Immunity

#### 2.3.1. Mechanisms Involving Cas Proteins and Direct Interaction with DNA

After CRISPR regions and *cas* genes were established to function in adaptive immunity in prokaryotes, Brouns and colleagues elucidated how Cas proteins work with the CRISPR loci in antiviral defense, published in August 2008 [38]. The small RNA sequences transcribed from the DNA sequences in CRISPR loci, termed CRISPR-RNA (crRNA), are generated by the action of a complex of Cas proteins. This complex was called the “CRISPR-associated complex for antiviral defense” or the CASCADE complex, comprised of five proteins, CasA, CasB, CasC, CasD, and CasE, in *E. coli* [38]. These proteins cleave the precursor crRNA to mature crRNAs, which are about 60 nucleotides long. These small, mature crRNAs act as small guide RNAs for the Cas protein complex to mount the antiviral response and prevent phages from infecting prokaryotes [38]. In December 2008, Carte and colleagues [39] used a recombinant protein, Cas6, one of the core proteins described earlier in 2005 [32] to show that Cas6 was an endoribonuclease that also cleaved precursor crRNA into small, mature crRNAs [39]. Subsequently, they confirmed this finding using natural Cas6 and identified the molecular interactions for this process [40]. 

Also in December 2008, another CRISPR mechanism was elucidated in a clinically isolated strain of *Staphylococcus epidermidis*. This bacterium, along with *Staphylococcus aureus*, is a common cause of nosocomial infections [41]. In these bacteria, horizontal transfer of genetic material in plasmids can occur by bacterial conjugation causing the recipient bacterium to acquire properties such as antibiotic resistance (reviewed in [42]). The strain studied contained a spacer region that was identical to a nickase (*nes*) gene found in all sequenced plasmids in *Staphylococci.* The authors showed that CRISPR loci interacted directly with DNA to cause interference and prevent the horizontal transfer of genetic material [41]. In 2009, the motifs adjacent to protospacers, which are only a few base pairs in length and had been observed earlier in *S. thermophilus*, were called PAMs (protospacer-adjacent motifs). PAMs were found to be conserved across multiple types of CRISPR arrays, and their precise sequence and location were found to be dependent on the type of CRISPR-Cas system [43].

Thus, by 2009, the following three stages of CRISPR mechanisms were described, as reviewed [44]:

1. Adaptation: the invading foreign DNA sequences, or the progenitor spacers (protospacers) are recognized by Cas proteins and those sequences are integrated into the host genome as spacers in the CRISPR loci.
2. Expression: the spacers integrated into the host genome are expressed as crRNA, which form a ribonucleoprotein unit with the Cas protein complex described above to process as single-guide RNA sequences.
3. Interference: the crRNA sequences cause interference with subsequently invading phage or plasmid genetic material to prevent the foreign genetic materials from infecting the host microorganism.

The next breakthrough came in 2010 when the exact molecular target of the CRISPR-Cas interference with the foreign DNA was discovered by Moineau in Quebec, Canada, and colleagues [45]. They showed that the CRISPR-Cas system in *S. thermophilus* used crRNA to directly target and precisely cleave the double-stranded DNA of viruses and plasmids, thus effectively preventing infection by phages and plasmids [45,46]. In 2010, Doudna and her group identified another Cas protein, Csy4, as an endonuclease that processes precursor crRNA to mature crRNAs in *Pseudomonas aeruginosa* [47]; subsequently, they showed that Csy4 is tightly bound to its substrate RNA with a very high affinity and detailed the mechanism of this highly specific interaction [48]. 

#### 2.3.2. Novel Mechanism: The Discovery of an Alternative Pathway of CRISPR Activation Using *trans*-Activating CRISPR RNA (tracrRNA)

In the meantime, Emmanuelle Charpentier, a microbiologist and scientist, was working with a mission to advance medicine [49]. She had begun to think about CRISPR in the early 2000s and wanted to understand every regulatory mechanism in the genome of the bacterium *Streptococcus pyogenes*. For this goal, she collaborated with Jörg Vogel, who was developing methods for the large-scale mapping of RNAs. When he mapped the entire small RNA sequences of *S. pyogenes* by 2008, they discovered a novel RNA that had not been previously identified [49]. They called it *trans*-activating CRISPR RNA or tracrRNA, thus identifying three components of the CRISPR system in *S. pyogenes*: crRNA, tracrRNA, and Cas proteins. 

At that time, three proteins, CasE, Cas6, and Csy4, had been shown to function as endoribonucleases for the processing of precursor crRNA to mature crRNAs, as described above [38,39,40,47]. Charpentier hypothesized that both types of RNA must work together in the CRISPR-Cas system to guide Cas endonucleases to the invading DNA virus, and a graduate student worked with her on the experiments required to prove her hypothesis [49]. The experiments worked, and their paper was published in March 2011 [50]. The function of tracrRNA was shown to process precursor crRNA (pre-crRNA) to mature crRNA in class II CRISPR-Cas systems that lacked the specific Cas genes *cse3 (CasE)*, *cas6*, or *csy4*, but contained *csn1* (*csn1* was later termed *Cas9*) [50]. 

#### 2.3.3. Reclassification of the CRISPR-Cas Proteins in 2011

In May 2011, Makarova and colleagues reclassified the CRISPR-Cas proteins based on the analyzed genomes of 639 bacterial and 67 archaeal taxonomic groups, to resolve the discrepancies that existed between the nomenclature of CRISPR-Cas systems and the Cas proteins [51]. The authors clarified in their paper the following example of confusing names [51]: the genes found in *E. coli* had been named earlier as *cse1*, *cse2*, *cse3*, *cse4*, and *cas5e* by Haft and colleagues [32], but these genes were also called *casA*, *casB*, *casE*, *casC*, and *casD*, respectively, by Brouns and colleagues [38]. At that time, three major types of CRISPR-Cas systems were described, Class I, II, and III, with their significant features shown in Table 1 [51]. In this new classification, Cas1 and Cas2 were present in all active CRISPR-Cas systems.

## 3. Development of CRISPR-Cas9 Single-Guide RNA-Mediated Gene Editing

In 2011, Charpentier met Jennifer Doudna, a biochemist, at a conference. The two scientists collaborated and published their discoveries in 2012 [1], leading them to receive the Nobel Prize in Chemistry in 2020 [52]. In their landmark paper, they built upon previous studies and identified the mechanisms of the type II CRISPR-Cas9 system in *S. pyogenes* for interference with invading DNA. They showed that the Cas9 endonuclease protein requires a dual-RNA-guided system comprised of tracrRNA and crRNA to target the double-stranded DNA [1]. Both nuclease domains of Cas9 were required for targeting the double-stranded DNA [1], consistent with findings in an earlier study wherein the CRISPR-Cas system from *S. thermophilus* was transferred into *E. coli* [53]. The precise site where the Cas9 endonuclease would act was determined both by the complementary base pair sequences in the protospacers and the protospacer adjacent motif (PAM) sequences [1]. In addition, they showed that the Cas9 protein could work with dual tracrRNA and crRNA-derived single-guide RNA sequences programmed to cleave specific sites in the target DNA, thus providing an alternative simple method for gene targeting and gene editing [1]. In a subsequent study published in January 2014, Doudna and colleagues showed the integral role of the trinucleotide PAM motif in the binding of Cas9 to target DNA and the precise cleavage of DNA [54]. 

Within months of their publication in 2012 [1], six papers were published in January 2013 demonstrating applications of CRISPR-Cas9 single-guide RNA-based gene editing, (reviewed in [2,55]). Three of these six papers showed gene editing in human cells [56,57,58]. After double-stranded DNA in human cells was cleaved by Cas9 single-guide RNA, the DNA was repaired by homologous recombination and non-homologous end joining [56,57,58]. Cas9, when mutated in one of the two nuclease domains, could be converted to a nicking enzyme that enabled a greater frequency of homolog-directed repair, reducing the effects of non-homologous end joining [56,57]. Multiple guide RNAs could be introduced simultaneously to edit several targets concurrently [56,57]. By 2014, numerous applications in diverse fields, including in medicine, had been developed using CRISPR-Cas9 single-guide RNAs, due to the versatile nature of this simple tool (reviewed in [3]). As early as 2013, a dominant *Crygc* mutation causing cataracts was corrected by CRISPR-Cas9 RNA-guided gene editing in a mouse model [59]. 

### Further Developments: Additional CRISPR-Cas Systems, Base Editing, and Prime Editing

In the decade since then, there have been innumerable advances in CRISPR-Cas RNA-guided gene-editing technology and applications in research, agriculture, and medicine, as described in many review articles; the interested reader is referred to the citations and references cited therein [4,5]. In 2014, the crystal structure of *S. pyogenes* Cas9 was described to comprise a recognition lobe and a nuclease lobe with two domains, with a groove at the interface to accommodate the heteroduplex of target DNA and sgRNA [60]. In 2014, Cas1 and Cas2 proteins were shown to function as a complex in vivo required for integrating spacers into CRISPR sequences [61], with Cas1 as the catalytic enzyme and spacer integration significantly enhanced by Cas2 [62]. In 2015 and beyond, additional CRISPR-Cas systems programmable by RNA-guided nucleases like *S. pyogenes* Cas9 were discovered by computational analysis, metagenomics [63], and experimental studies to add to the CRISPR-Cas9 toolkit for genome editing (Zetsche et al., 2015 [64], Schmakov et al., 2015 [65], Abudayyeh et al., 2016 [66], East-Seletsky et al., 2016 [67], Burstein et al., 2017 [68], Schmakov et al., 2017 [69], Koonin et al., 2017 [70], Yan et al., 2018 [71], Harrington et al., 2018 [72], Yan et al., 2019 [73]). These additions included Cas9 homologs, Cas12a, Cas12b, Cas12d, and Cas12e, which target double-stranded DNA, and Cas13a and Cas13b, which target single-stranded RNA (reviewed in [4,74]). In 2015, the CRISPR-Cas systems were classified into 2 major classes, 5 types (Types I, II, III, IV, and V), and 16 subtypes [75]. The classification was updated in 2020 to include 2 classes, 6 types (added Type VI), and 33 subtypes [76] and is summarized in Table 2. A complex of multiple proteins characterizes the effector proteins in Class 1, whereas Class 2 proteins consist of a single, large unit [75,76]. Cas9, Cas12, and Cas13 are examples of Class 2 proteins. Cas9 is a type II Cas protein, and Cas12 and Cas13 belong to types V and VI CRISPR-Cas systems, respectively (reviewed in [74]). Types II and V in Class 2 are fundamentally different in the structural domains of the effector proteins. In the Type II effector protein, Cas9, each of the two nuclease domains, one RuvC-like and the other HNH domain, cleave one strand of the target double-stranded DNA. In contrast, there is only one RuvC-like nuclease domain in Type V Cas12 effectors, which cleaves both DNA strands [76]. Type VI Cas 13 proteins are comprised of two Higher Eukaryotes and Prokaryotes Nucleotide-binding (HEPN) domains [76,77].

In humans, double-stranded DNA breaks occur for physiological processes such as V-(D)-J rearrangements and class switch recombination in B-lymphoid cells. These DNA breaks are repaired by non-homologous end joining, which is the primary manner for double-stranded DNA repair physiologically [78]. Therefore, the cleavage of double-stranded DNA by Cas nucleases in human cells triggers DNA repair by non-homologous end joining, which is more efficient than homology-directed repair. However, non-homologous end joining leads to insertions and deletions to repair that DNA strand, which is undesirable for genetic editing since it could lead to irreversible pathogenic consequences. Therefore, homology-directed repair is preferred for genetic editing, but this process requires donor DNA to be introduced and this repair mechanism is active only during the synthetic S/G2 phase of the cell cycle. For details of these two and other recently identified DNA repair processes in the context of CRISPR-Cas9 gene editing, the reader is referred to the cited reviews [79,80]. 

Meanwhile, in 2016, David Liu and his group developed base editors that directly and irreversibly converted one nucleotide base into another base without cutting the double-stranded DNA strand or requiring a donor template [81]. They engineered a programmable enzyme similar to CRISPR-Cas by fusing Cas with a cytidine deaminase enzyme to directly convert cytidine to uridine, or a C to U conversion, leading to a C to T, or a G to A, base substitution. Thus, this method avoided the need to repair double-stranded DNA for CRISPR-Cas-RNA-based gene-editing systems and could be applied to genetic diseases with a point mutation [81]. However, this method only converted C-G to T-A, representing half of the possible single nucleotide variants. Therefore, they devised another method for base editing that converted A-T to G-C, termed adenine base editors, published in 2017 [82]. Then, all four transitions, C to T, A to G, T to C, and G to A, were possible by the two base-editing methods [81,82]. 

Nevertheless, the eight transversion conversions, C to A, C to G, G to C, G to T, A to C, A to T, T to A, and T to G, targeted deletions or insertions that were not possible by base editing. These mutations comprise about 70% of the reported pathogenic mutations in human diseases, with transition mutations comprising 30% [83]. Therefore, Liu and his group engineered “prime editors”, published in 2019, which also did not need the repair of double-stranded DNA or donor DNA templates [83]. They described this advance as a “search and replace” method, which could edit all 12 possible point mutations, targeted deletions, and insertions, in any combination in human cells [83]. Many subsequent advances in gene-editing technology were described in a subsequent review, including minimizing off-target gene-editing activity, considerations for which editing application to select for specific genomic edits, and the requirements and challenges ahead to continue to innovate and engage all stakeholders to achieve the full potential to benefit society [84]. Further continuous developments in advancing the efficiency of prime editing by Liu’s group were published in 2021 [85] and 2023 [86]. Prime editing was described as “a super-precise new CRISPR tool for tackling genetic diseases” in 2019 [87]. Base editing and prime editing were discussed as examples of the “next generation of CRISPR-Cas technologies” in 2019 [88], “CRISPR genome editing” in 2022 [89], and “CRISPR 2.0” in 2023 [90].

## 4. Hematopoietic Stem Cell Transplantation with Gene-Edited Hematopoietic Stem Cells

Hematopoietic stem cell transplantation is an established treatment for many hematologic diseases, including leukemias, lymphomas, multiple myeloma, aplastic anemia, and genetic diseases such as sickle cell anemia, thalassemia, severe combined immunodeficiency, Wiskott–Aldrich syndrome, Fanconi anemia, other inherited bone marrow failure syndromes, and inborn errors of metabolism [8]. 

The history and the process of performing autologous HSCTs with genetically edited stem cells, i.e., gene therapy, was reviewed comprehensively in 2017; the reader is referred to this excellent review article describing the progress over the last 30 years and the lessons learned [9]. Allogeneic HSCTs have been used in the following disease groups: (1) primary immune diseases, including severe combined immunodeficiency (adenosine deaminase deficient, X-linked, and other genetic forms), Wiskott–Aldrich syndrome, chronic granulomatous disease, leucocyte adhesion deficiency, hemophagocytic lymphohistiocytosis, X-linked hyper IgM syndrome, X-linked lymphoproliferative disease, and X-linked agammaglobulinemia; (2) hemoglobinopathies such as sickle cell disease and β-thalassemia; (3) storage and metabolic disorders, including Gaucher disease and other lipidoses, mucopolysaccharidoses, X-linked adrenoleukodystrophy, metachromatic leukodystrophy, and osteopetrosis; and (4) congenital cytopenias and stem cell defects such as Fanconi anemia, Schwachman-Diamond syndrome and severe congenital neutropenia (also called Kostmann syndrome) [9]. Gene therapy using γ-retroviral, lentiviral, or both types of viral vectors, including preclinical stages, has been applied in at least 17 diseases in the above groups, as described in the review in 2017 [9]. The authors noted that developing hematopoietic stem cell gene therapy took a long time, almost 30 years for clinical gene therapy to advance from when the methods were developed for gene transfer by viral vectors in the 1980s [9]. Another excellent review in 2020 showed the timeline of gene therapy from the 1980s until 2020 [91]. In 2020, Rai and colleagues found that CRISPR-based genetic editing in Wiskott–Aldrich syndrome was highly effective and safer than lentivirus-based gene editing [92]. In 2022, lentiviral gene therapy was reported to be effective in a study of ten infant patients with newly diagnosed Artemis-deficient severe combined immunodeficiency, a disease caused by mutations in the *DCLRE1C* (DNA cross-link repair 1C) gene, for which allogeneic hematopoietic stem cell transplantation is not helpful [93]. 

Hematopoietic stem cell (HSC) gene therapy requires the collection of autologous HSCs to edit them genetically ex vivo, followed by transfusion of the edited stem cells back into the same patient [9,91]. The collected HSCs are usually enriched for CD34+ cells since the multipotent progenitor cells needed for long-term engraftment are present in the CD34+ compartment of the bone marrow and comprise about 1% of bone marrow cells. After engraftment, these long-term HSCs divide multiple times to form normal hematopoietic cells. Therefore, successful gene therapy requires that the genetic edits be permanent in the HSCs and be transmitted to the daughter cells when the HSCs divide multiple times. In addition, the long-term progenitor cell capacity must be retained, and there should be no adverse effects [9]. 

Clinical trials for gene therapy were initially conducted by academic researchers, as described [9]. A publication entitled “Gene Editing Pipeline Takes Off” in 2020 showed a timeline of clinical trials supported by the industry for gene therapy [94]. Zinc finger nucleases, termed ZFNs, were the first gene editors used in clinical trials, followed by transcription activator-like effector nucleases (TALENs) and CRISPR-Cas9 in 2016 [94]. ZFNs were developed in the early 2000s [95], followed by TALENs in 2011 [96,97]. A ZFN is a genetically engineered endonuclease that targets zinc finger proteins in the genome [95]. It is constructed by fusing a zinc finger protein to the cleavage domain of a unique Class II restriction enzyme, *Fok*I, derived from *Flavobacterium okeanokoites* [95,98]. *Fok*I must dimerize to cleave DNA and requires two DNA binding sites, which leads to its specificity to recognize 18–36 base pair (bp) sequences [95]. By 2010, three clinical trials had been started using ZFNs [95]. A timeline of key publications from 1989 to 2011 related to the development of TALENs was published in 2011 [96]. Transcription activator-like effector (TALE) proteins are natural proteins produced by *Xanthomonas* species, which are pathogenic to plants. These proteins enter the plant cells and bind with the host DNA to colonize the host. TALE proteins contain repeats of 34 amino acids, of which the two at positions 12 and 13 are specific for DNA binding. Like ZFNs, TALE proteins are fused with *Fok*I for the endonuclease activity [95,97].

After 2012, the simplicity and versatile nature of CRISPR-Cas9 guide-RNA-based gene editing over the existing gene-editing methods, as described by Doudna and Charpentier, led to its numerous and rapid applications in clinical research, briefly described next.

## 5. Clinical Applications of CRISPR-Cas-Based Gene Editing

### 5.1. Ethical Considerations: Meeting in Napa, California

In 2015, only three years after their landmark paper in 2012, Doudna described her whirlwind year with the ethics of gene editing, after being astounded by the “breakneck pace” with which the discovery was being applied worldwide to diverse fields, including agriculture and medicine [99]. There were questions described in that publication that she had received from society, including one from a woman diagnosed with carrying a germline *BRCA1* mutation, which is known to predispose to breast and ovarian cancer [99]. The author described how her experience that year had led her to form a group that met urgently in Napa, California, to develop guidelines for responsible and ethical ways of applying gene editing in humans [99], which were published in March 2015 [100]. For clarification, the terms “genome modification” and “germline engineering” refer to changes in the DNA of the nucleus of a germ cell, as described in the publication [100]. The discussions about the scientific, medical, legal, and ethical implications of genome editing led the group to “strongly discourage any attempts at germline genome modification for clinical application in humans” [100]. They also noted that such activity was “illegal or tightly regulated in countries with a highly developed bioscience capacity” [100].

### 5.2. Selected Clinical Research Applications

Numerous published examples of clinical research are available by searching the literature via the PubMed database for applications of the CRISPR-Cas-based gene-editing technology in the last decade, including infectious disease diagnostics, screening by base editors for genetic variants, mouse models of various human genetic diseases, and examples of treating genetic diseases. The base editors described above had entered the clinic by 2022, including in sickle cell disease [101,102]. A publication in March 2023 listed 23 clinical trials using CRISPR-Cas9-based gene therapy for editing hematopoietic stem cells [103]. These included 4 clinical trials for sickle cell disease, 4 for β-thalassemia, 5 for leukemias, 1 for non-Hodgkin lymphoma, 1 for multiple myeloma, 2 for human immunodeficiency virus, and 6 for non-hematologic tumors [103]. 

#### 5.2.1. Chimeric Antigen Receptor (CAR)-T Cell Therapies in Hematologic Malignancies

Chimeric antigen receptor (CAR)-T cells contain a synthetic receptor directed against a specific antigen present on the surface of the cancer cells, which allows the CAR-T cells to target the cancer cells without engaging the major histocompatibility complex [104]. Several CAR-T cell therapies directed against the surface antigens CD19, and B-lymphoid cell maturation antigen (BCMA) have been approved for treating patients with relapsed or refractory B-lymphoid cell malignancies, including leukemias, lymphomas, and multiple myeloma (reviewed in [105]). These novel immunotherapies have shown unprecedented treatment responses in B lymphoblastic leukemia, B-cell lymphomas/leukemias, and multiple myeloma. For further progress, combining CAR-T cell therapies with the advances in CRISPR-based genetic editing has already led to promising clinical research toward treating patients with all hematologic malignancies, including T-cell neoplasms and acute myeloid leukemia. 

In June 2023, results were published from a phase I clinical trial of CRISPR base-edited chimeric antigen receptor (CAR)-T cells specific for the CD7 antigen, which is present on the surface of T lymphoblasts, in treating the first three patients with relapsed T-cell acute lymphoblastic leukemia, which is a very aggressive T-lymphoid cell malignancy. In contrast with the approved CAR-T cell therapies for B-cell malignancies, these study investigators obtained normal T cells from healthy donors. They first edited the DNA in the T cells by CRISPR base editing to disrupt the CD7 antigen and αβ T-cell receptors in the T cells to prevent CAR-T cell death and graft-versus-host disease, respectively, after the therapy. These gene-edited healthy T cells were then used to generate the CAR-T cells specific for CD7. Of the three patients who received the gene-edited CAR-T cell therapy, two achieved remission and one died of infection as a known complication of CAR-T cell therapies [106]. 

In September 2023, three publications described CRISPR-based gene-edited approaches in developing CAR-T cell therapies in hematolymphoid neoplasms [107,108,109], including AML and related neoplasms [107], which are aggressive hematologic malignancies with still-unmet treatment needs for many patients with relapsed or refractory leukemia. These three studies used base editing to edit the antigens on the surface of hematopoietic stem cells that were to be used as targets in the CAR-T cells so that the leukemic cells would be targeted but the engrafted hematopoietic stem cells would escape destruction and retain their long-term stem cell properties to preserve hematopoiesis. Casirati and colleagues [107] focused on AML and generated epitope-engineered HSCs with base edits for point mutations in *FLT3* or CD135, the α-subunit of IL-2 receptor or CD123 [110], and *KIT* or CD117, including by multiplexing these edits. They constructed CARs with specificity for the same epitopes, which are commonly present on the surface of AML cells. They confirmed the effectiveness of this approach in patient-derived AML xenografts, including eradicating leukemic cells and preserving hematopoietic cells [107]. Marone and colleagues experimentally showed that editing the gene encoding CD123 in hematopoietic stem and progenitor cells (HSPCs) protected the HSPCs from CD123-targeted immunotherapy and preserved their function [109]. 

Wellhausen and colleagues [108] focused on developing a universal CAR-T cell therapy and base-edited CD45, a pan-leukocyte antigen, in hematopoietic stem cells, so that the CAR-T cells could target CD45 on the cancer cells, but the engrafted hematopoietic stem cells and the CAR-T cells would not be killed. They inserted a non-synonymous mutation in the T cells in the gene encoding CD45 so that the CD45 function was retained, and the T cells were not recognized by the anti-CD45 clone. The single amino acid substitution was sufficient for their intended purposes of leukemia eradication and preserving normal cell functions [108], similar to a single point mutation being sufficient in the previous study [107]. 

#### 5.2.2. Ex Vivo Adenine Base Editor Gene Therapy in a Primary Immunodeficiency Disorder

In 2023, preclinical studies conducted by McAuley and colleagues [111] toward curing CD3δ severe combined immunodeficiency showed that adenine base editing of HSPCs effectively converted the disease’s pathogenic point mutation c.202C>T in *CD3D* in a cell line-based study and lentivirus-induced pathogenic mutations in healthy human CD34+ cells’ disease models, and in CD34+ HSPCs from a patient with the disease [111]. Then, the investigators showed that the edited HSPCs could produce functional T cells with a normal T-cell repertoire in an artificial thymic organoid model, thus showing ex vivo base editing as a promising non-viral, non-double-stranded DNA repair strategy for curing these patients [111].

#### 5.2.3. In Vivo CRISPR-Based Gene Editing, Including Editing Hematopoietic Stem Cells

In 2021, promising results were published from a phase I, in vivo, CRISPR-Cas9-based gene-editing clinical trial in transthyretin (TTR) amyloidosis, in which progressive potentially fatal damage occurs due to the deposition of misfolded TTR protein in nerves and the heart. The in vivo aspect was achieved by lipid nanoparticles with hepatic specificity to deliver mRNA for Cas9 and single-guide RNA for TTR to hepatocytes [112]. 

An exciting development in 2023 was the in vivo delivery of a base-editing system comprised of a lipid nanoparticle containing mRNA directed against the stem cell factor receptor, CD117, on the surface of hematopoietic stem cells in the bone marrow of mice [113]. The investigators showed that the lipid nanoparticle-based editing system could carry diverse mRNA and successfully modify hematopoietic stem cells. They also showed that the delivery of a “genetic medicine” in the form of pro-apoptotic PUMA (p53 up-regulated modulator of apoptosis) mRNA in the lipid nanoparticle could avoid the toxic conditioning required to kill the pre-existing bone marrow cells in preparation for a hematopoietic stem cell transplant. Thus, this highly innovative method successfully edited HSCs in vivo and with non-genotoxic conditioning, which patients with Fanconi anemia and other similar diseases could also benefit from [113]. 

#### 5.2.4. Sickle Cell Disease and Transfusion-Dependent β-Thalassemia

Sickle cell disease and thalassemias are common inherited monogenic disorders of hemoglobin with a worldwide prevalence. Sickle cell disease is diagnosed in about 300,000 newborns annually, with the highest incidence in sub-Saharan Africa, and India, and a prevalence of about 100,000 affected individuals in the USA [114]. Individuals with sickle cell anemia have a single point mutation in the hemoglobin B (*HBB*) gene causing the amino acid at codon 6 to change from glutamine to valine in both alleles, i.e., they are homozygous for the sickle hemoglobin gene, *HbS.* The disease is characterized by vaso-occlusive crises due to the blocking of small blood vessels by the sickled hemoglobin in red blood cells, leading to acute pain and organ failure. Chronic complications occur due to large-vessel disease and progressive ischemic organ damage [114].

β-thalassemia is an inherited hematologic disease characterized by a quantitative defect in the β-globin chain of hemoglobin leading to ineffective erythropoiesis and hemolysis. The reduction in β-globin chains leads to a skewed ratio of α- and β-globin chains with the excess α-chains forming tetramers that cause cellular dysfunction and cytoskeletal damage. β-thalassemia is estimated to occur in about 1.5% of the world’s population, with 90% of cases occurring in the tropical “thalassemia belt”, including sub-Saharan Africa, the Middle East, the Indian subcontinent, and southeast Asia [115]. Patients with β-thalassemia major require blood transfusions starting early in life within 2 years of age and are transfusion-dependent for life until an expected age in the sixth decade. Therefore, both sickle cell disease and transfusion-dependent β-thalassemia are chronic debilitating diseases. 

On December 8, 2023, the United States Food and Drug Administration (FDA) approved the first CRISPR-Cas9 RNA-based gene therapy, exagamglogene autotemcel (exa-cel; Casgevy^TM^), and a ZFN-based gene therapy (Lyfgenia^TM^) to treat patients aged 12 years or older with severe sickle cell anemia [18]. Patients receiving the ZNF-based therapy [116] have developed hematologic cancer [18,117], and therefore, patients receiving this therapy must receive lifelong surveillance for hematologic cancers [18,117]. In January 2024, the CRISPR-based treatment was also approved by the FDA for treating patients 12 years or older with transfusion-dependent β-thalassemia. The CRISPR-based gene therapy works in both diseases due to an edited *BCL11A* gene, which is normally repressed at birth causing a switch from fetal to adult hemoglobin. The genetic editing of *BCL11A* reactivates the gene to become functional again to produce fetal hemoglobin (reviewed in [118]). The study investigators enrolled 44 patients aged 12–35 years with severe sickle cell anemia and 52 patients aged 12–35 years with transfusion-dependent thalassemia in an open-label multi-institutional international trial. All sickle cell disease patients had a history of at least two severe vaso-occlusive crises in the two years before enrollment. The results in sickle cell anemia showed that 29 (93.5%) of 31 patients did not have a severe vaso-occlusive crisis in 12 months after receiving the therapy, and 30 (100%) of 30 did not need hospitalization in the 12 months after treatment. In total, 32 (91.4%) of 35 patients with β-thalassemia did not need a transfusion for at least 12 months after receiving the therapy [119].

These approvals for a one-time infusion therapy designed to last permanently represented a landmark for the scientific community and the field of medicine to benefit patients in a transformative manner. The first U.S. patient with sickle cell disease who was treated with this therapy described her experience at an overseas medical conference as a “miracle that allowed her to start living her life and do things she had never been able to do before due to her disease” [120]. Other gene therapies are also being developed for these diseases, using different approaches [121,122,123,124,125].

#### 5.2.5. First Clinical Trial Using Prime Editing Gene Therapy: Chronic Granulomatous Disease

During the preparation of this manuscript, the U.S. FDA approved the first prime editing clinical trial in humans with chronic granulomatous disease on 29 April 2024 [126]. This milestone paves the way for many more possible permanent gene therapy treatments for numerous patients with other diseases, including inherited germline predisposition to hematologic malignancies, discussed next.

## 6. Known Inherited Germline Predispositions to Hematologic Malignancies

Germline predispositions to malignancy are now established as being present in many patients with hematolymphoid neoplasms. These predispositions to developing a neoplasm occur due to genetic mutations in germline tissues. Germline mutations may be inherited from a parent or occur de novo, i.e., without being inherited from a parent. Many genes critical to the proper functioning of hematopoietic systems and cellular pathways may be affected by germline mutations. Some of these inherited germline mutations cause inherited diseases such as bone marrow failure syndromes. Other genetic mutations may manifest without syndromic features. Any of these germline mutations, including those in inherited bone marrow failure syndromes, may be present even without a family history or clinical features typically associated with the disease. Inherited bone marrow failure diseases are more common in the pediatric age group but may also be diagnosed in adults. Similarly, genetic tumor syndromes and familial AML and myelodysplastic syndromes may manifest with disease in the pediatric or adult ages, with some age predilections such as germline *GATA2*, *SAMD9*, and *SAMD9L* abnormalities often being identified in myeloid neoplasms presenting in the pediatric age group, and germline *DDX41* also presenting in older adults. The spectrum of germline mutations that predispose to cancer is vast, continuously being discovered, and is associated with a tremendous heterogeneity in clinical manifestations, including a variable penetrance of the disease among different families and even within the same family with members carrying the same germline mutation (reviewed in [20,21]).

### 6.1. Diseases with Germline Predisposition to Hematologic Malignancies in the Pediatric and Adult Age Groups:

Table 3 summarizes the most well-known genetic diseases with an inherited or de novo germline predisposition to hematolymphoid malignancies, including the most commonly identified defective genes, the defective function or cellular pathways due to the genetic defect, and selected references for each condition.

An allogeneic HSCT is the only way to cure the diseases shown in Table 3. These diseases represent a broad spectrum with an underlying germline genetic abnormality, each requiring individualized management. Among these diseases, efforts to develop lentiviral-based gene therapy have been directed mainly toward treating inherited bone marrow failure syndromes. The diseases in the category of familial AML and myelodysplastic neoplasm shown in Table 3 were described only in the last two decades, with increasing recognition of cancer-predisposing germline genetic defects due to the increased use of next-generation sequencing for the diagnosis of these hematopoietic neoplasms. Donor-derived leukemia due to any cause is another grave complication of an allogeneic HSCT, and this occurrence is considered to be underdiagnosed and mistaken for relapsed leukemia (reviewed in [184]). Of note, when an allogeneic HSCT is offered to a patient with familial leukemia or myelodysplastic neoplasm, there is a risk of having an HLA-matched familial donor also having the same germline mutation as in the patient being treated. Several instances of these donor-derived leukemias have been reported, including from the donor harboring a germline *CEBPA* mutation [185] and germline *DDX41* mutations [186,187,188].

### 6.2. Gene Therapy Applications in Inherited Bone Marrow Syndromes

Patients with inherited bone marrow failure syndromes may be potentially cured by an allogeneic HSCT, with improved results following lower toxicity protocols in Fanconi anemia (reviewed in [189]). However, an allogeneic HSCT is not always offered due to its potential complications, as reviewed in November 2023 [190]. In 2019, lentiviral-mediated autologous gene therapy showed promising results, including stopping the progression of bone marrow failure in patients with Fanconi anemia harboring germline *FANCA* mutations [191]. Following the clinical studies in Fanconi anemia in 2019 [191], preclinical studies reported in May 2024 showed that lentivirus-mediated genetically edited CD34+ hematopoietic stem cells in Diamond–Blackfan anemia improved erythroid maturation [192]. 

Preclinical studies toward treating Fanconi anemia have been performed using adenine base editors and sgRNA in patient-derived and healthy donor-derived lymphoblastoid cell lines and CD34+ hematopoietic stem cells from healthy donors and patients with Fanconi anemia [193]. The authors [193] investigated the effects of two adenine base editing systems, including the newer one developed in 2020 [194] in correcting a single point mutation in *FANCA*. They found that the newer adenine base editor had a higher efficiency in base editing than the previous version [193]. The most promising preclinical development for Fanconi anemia and other disorders with defects in DNA damage response and DNA repair appears to be the delivery of a lipid nanoparticle for the base editing of hematopoietic stem cells in vivo, as described by Breda and colleagues in 2023 [113].

## 7. Why Select CRISPR-Based Gene Editing to Treat Individuals with an Inherited or Germline Predisposition to Hematologic Cancers?

As stated earlier, germline predispositions to hematologic malignancies are heterogeneous, and there is variable disease penetrance even within a family with the same germline predisposition to cancer. In addition, there may be individuals who carry the germline mutation but never develop the disease, whereas another member in the same family does. Consequently, an inherited predisposition to hematologic malignancies is often diagnosed after the first patient in a family develops a neoplasm, most often a myelodysplastic neoplasm or AML, which may require an allogeneic HSCT for treatment. While carriers of a pathogenic germline mutation who do develop AML or another malignancy are treated for the malignancy, there is currently no treatment for the germline mutation itself. Family members of the proband are offered genetic testing and counseling after a proband is found to have a germline predisposition, but there is no treatment available other than monitoring the carrier individual at regular intervals until the disease, if it is meant to progress, manifests clinically. It is also worth noting that in all situations where germline testing is performed, informed consent and genetic counseling are required.

It should be noted that considering gene therapies in this heterogenous population of individuals with germline predispositions to cancer would have to consider the following separate groups of carriers, which are discussed in the subsequent sections, and with examples applicable to any of the germline predispositions to cancer given in Table 3:

1. Individual carriers of germline predisposition to cancer who have developed a hematologic malignancy such as AML or myelodysplastic neoplasm or any other hematolymphoid malignancy associated with that germline defect.
2. Family members of a proband diagnosed with a malignancy and carrying the germline predisposition, but the related members are healthy and have not developed any malignancy.
3. Potential donors for an allogeneic HSCT to be given to a patient with a germline predisposition with developed cancer such as AML, including related (familial) or unrelated donors.

Currently, a patient who has progressed to a malignancy such as AML is often offered an allogeneic HSCT. The only available treatment for carrier individuals with a high risk of transforming to malignancy is also an allogeneic HSCT, with its associated morbidity and even mortality. As mentioned, in the situations where an allogeneic HSCT is currently offered to individuals with a germline predisposition, CRISPR-based genetic editing of HSCs would allow an autologous HSCT with genetically modified HSCs, abrogating the potentially life-threatening complications of an allogeneic HSCT, including eliminating the risks of both graft-versus-host disease and donor-derived leukemia. Further, base-editing and prime-editing technologies do not create a double-stranded DNA break, which is especially valuable in any disease with an underlying DNA repair defect. 

## 8. When Could Patients Be Considered for CRISPR-Based Gene Editing to Treat Individuals with an Inherited or Germline Predisposition to Hematologic Cancers?

The most obvious time point for a patient with a germline predisposition to a malignancy to receive a CRISPR-based gene-edited autologous HSCT would be at the same time when an allogeneic HSCT would be currently planned for or offered to that patient. These situations could be broadly grouped as follows:

1. Individuals diagnosed with an aggressive hematologic malignancy such as AML and found to have an underlying germline genetic predisposition to malignancy are often offered an allogeneic HSCT. For example, AML with germline *CEBPA* mutations, included in Table 3 as the first entry in the familial AML and myelodysplastic neoplasm group, has a natural history of relapsed AML, which can only be treated to prevent future relapses by an allogeneic HSCT [163]. An autologous HSCT with gene-edited hematopoietic stem cells in individuals carrying these germline mutations, if performed in complete remission after the first occurrence of AML, would cure these individuals of their germline predisposition and prevent relapsed AML.
2. Defects in the other genes shown in Table 3 in this same group predispose to both myelodysplastic neoplasms and AML, and similar considerations as in AML with germline mutated *CEBPA* described above could be applied to developing gene therapies in individuals in each of these groups after the development of a hematologic malignancy.
3. Other individuals carrying a pathogenic germline mutation and identified clinically as having a high risk of developing a malignancy, in whom a pre-emptive allogeneic HSCT is currently attempted before the development of other complications such as in *GATA2* germline abnormalities, may also benefit from CRISPR-based gene-edited autologous HSCT.
4. Further, gene-edited hematopoietic stem cells from an HLA-matched familial donor could be used for a matched donor allogeneic HSCT for a patient with a hematologic malignancy carrying a familial germline mutation, to prevent the possibility of transmitting a leukemic predisposition through the donor’s transplanted cells and eliminating the possibility of a donor-derived leukemia in the recipient.

## 9. Which Patients with an Inherited or Germline Predisposition to Hematologic Cancers Could Be Selected for CRISPR-Based Gene Editing?

1. All patients with inherited bone marrow failure syndromes would benefit since bone marrow failure is a serious condition, and if progressive bone marrow failure could be prevented by a CRISPR-based genetically edited autologous transplant, that would be a huge breakthrough for these patients.
2. Most diseases in the group with inherited tumor syndromes shown in Table 3 would also benefit from this treatment when developed. For example, patients with Li–Fraumeni syndrome have a high lifetime risk of developing cancer, which can even include multiple types of cancer, and these patients and family members undergo a lifetime of surveillance for cancer in various body sites. The cancers arise due to the loss of the tumor suppressor function of TP53, the protein encoded by the *TP53* gene, which normally preserves genomic stability by acting on many downstream targets (see cited references [195,196,197,198,199]). Germline defects in *TP53* in Li-Fraumeni syndrome are often missense mutations. The toughest part of developing any gene therapy to treat or cure this hereditary disease would be the fact that the *TP53* mutations in this disease are present in many non-hematologic tissues. Nonetheless, with so many technological advances in gene editing and multiple clinical trials using base editing currently in progress in humans, and showing clinical results unimaginable even a decade ago, including in treating inherited diseases of the eye and retina, the author hopes that worldwide experts in the field will design preclinical studies to bring to clinical studies eventually.
3. In the group with familial AML and myelodysplastic neoplasms shown in Table 3, despite much progress in the last decade in our understanding of these genetic predispositions to AML and myelodysplastic neoplasms, it is not yet known throughout this genetic spectrum which individuals harboring a germline predisposition are at the highest risk for developing a malignancy. Inherited thrombocytopenias due to *RUNX1*, *ANKRD26*, or *ETV6* germline mutations have variable disease penetrance, with a maximum of 40–60%, and highly variable inter-familial and intra-familial clinical features that may be mild to severe. The risk of developing a malignancy is often identified only after the individual carrying a pathogenic germline mutation develops a hematologic malignancy. Therefore, as described above, patients who need an allogeneic HSCT could benefit from a CRISPR-based gene-editing approach, which requires study.

In this group, it is easier to visualize offering an autologous HSCT with gene-edited hematopoietic stem cells in the future to those carriers with a germline predisposition who have already manifested clinically with a hematologic malignancy, as discussed above. It is much more difficult to envision when gene therapy could be offered to carriers of pathogenic germline mutations who have not yet developed a malignancy, to prevent the development of the first clinical manifestation of a hematologic malignancy. The most important reason for this current inability is that every carrier individual with a pathogenic germline mutation does not necessarily develop a malignancy, and as mentioned earlier, who will develop malignancy and who will not, is yet to be understood. These very important questions, when answered, could provide an answer for when gene therapy could be offered to prevent the development of malignancy in those carriers with germline predisposition who would eventually develop a malignancy. 

Nevertheless, germline *DDX41* abnormalities are worthy of further thought and discussion. *DDX41* is the most common gene affected by pathogenic germline abnormalities, as learned from several extensive studies published since 2015 (reviewed in [20]). These germline abnormalities are identified in older adults in the same age group as sporadic AML and myelodysplastic neoplasms and are more common in males. In addition, the UK Biobank study showed that these germline mutations are quite common in the general population, making it possible that a germline *DDX41* abnormality could be present in any donor for an allogeneic HSCT, even without a family history of germline mutations [175]. Ideally, developing a universal CRISPR-based strategy in the future to potentially eliminate the possibility of transmitting a donor-derived *DDX41* germline mutation to a transplant recipient would greatly help patients. To achieve this goal, ethical and financial considerations would need to be addressed, including who would pay for the testing, including pre-test and post-test genetic counseling, and what treatment, if any, the donor would receive if found to be a carrier of a pathogenic germline *DDX41* mutation. These sensitive aspects related to germline predisposition and allogeneic HSCT are beginning to be discussed in the UK [200].

In the pediatric age group, germline abnormalities in *GATA2*, *SAMD9*, and *SAMD9L* are the most common causes of myelodysplastic neoplasms and AML. These abnormalities can be inherited or de novo, with diverse complications involving multiple organ systems. The only treatment is an allogeneic HSCT, which has been reported to have worse outcomes in patients with germline *GATA2* mutations than in patients without such abnormalities [201]. CRISPR-based gene therapy should also be studied for patients with these pathogenic germline mutations to see if the results are better than allogeneic hematopoietic stem cell transplantation.

## 10. Conclusions

The quest for scientific discovery, innovation, and the motivation to advance medicine has led to progressive transformational changes in the landscape of treating patients with various diseases, most prominently genetic diseases that had no effective treatments until gene-therapy studies were initiated. The entire journey of the discovery and understanding of CRISPR alone and its application in gene editing is highly instructive for any future advances and a testament to what can be achieved with worldwide collaboration. It took 25 years from a repetitive sequence of unknown significance in a bacterium in 1987 to the 2012 discovery of the CRISPR-Cas single-guide RNA gene targeting and editing. In less than 12 years since then, building on the lessons learned from earlier gene therapy efforts, biotechnology has advanced tremendously to transform the landscape in medicine to enable one-time permanent treatments by giving a single “gene therapy” dose, with sickle cell disease being the first example. As mentioned earlier, these breakthroughs combined with other advances, including chimeric antigen receptor T cell therapies, have the potential to effectively cure many aggressive hematologic diseases in a manner that was unimaginable even a decade ago. This revolution in patient care has a real potential to transform the lives of numerous patients afflicted with genetic abnormalities, particularly individuals with aggressive hematologic malignancies with underlying germline genetic abnormalities. Given the current transformational landscape in gene editing, studies to apply CRISPR-based gene editing toward gene therapy in individuals with these conditions must be prioritized.

## Figures and Tables

**Table 1:** The signature genes, encoded proteins, and functions known in the three classes of CRISPR-Cas systems in 2011 (Makarova et al., 2011 [51]).

CRISPR-Cas Systems	Genes	Encoded Proteins	Functions
Type I (Bacteria and Archaea) included 6 subtypes	Signature: *cas3*	Large protein with helicase and DNase activity	DNA target cleaved by the HD endonuclease domain of Cas3
	Other genes	Cascade complex proteins, including Cas5, Cas6, Cas7, and Cas8 in the RAMP superfamily	Two subtypes of Cas6 cleave pre-crRNA to generate crRNAs
Type II (exclusively in Bacteria until 2011) included 2 subtypes	Signature: *cas9*	Single, large protein with two nuclease domains: a RuvC-like domain near the amino-terminus and an HNH domain in the middle of the protein	Cleave pre-crRNA by dual *trans*-activating RNA and part of the repeat in the pre-crRNA [50]
Type III (more common in Archaea) included 2 subtypes	Signature: *cas10*	RAMP proteins	Mediate DNA cleavage
Unclassified loci	Signature gene not identified; not classifiable as any Type		

Abbreviations: RAMP, repair-associated mysterious protein; pre-crRNA, precursor CRISPR RNA; crRNA, CRISPR RNA.

**Table 2:** Summary of the classes, types, and subtypes of CRISPR-Cas systems in 2020 [75,76].

CRISPR-Cas Systems	Types	Hallmarks of Types of CRISPR-Cas Systems	Subtypes	Targets
Class 1	Type I	Cas3	I-A, I-B, I-C, I-D, I-E (I-E of *E. coli* is best characterized), I-F1, I-F2, I-F3, I-G *	DNA
	Type III	Cas10	III-A (usually contain *cas1*, *cas2*, and *cas6*), III-B, III-C, III-D, III-E, III-F	DNA and RNA
	Type IV (putative in 2015)	Genes resembling *cas5*, *cas7*, and *cas8*	IV-A, IV-B, IV-C	
Class 2	Type II	Cas9; *cas9* in the vicinity of *cas1* and *cas2* genes	II-A: also contain *csn2* gene; II-B: also contain *cas4* gene; lack *csn2* gene; II-C: contain only *cas1*, *cas2*, and *cas9*; the most common system in bacteria, including II-C1, II-C2	DNA
	Type V	Cas12	V-A: Cas12a (Cpf1); V-B: Cas12b (C2c1); V-C: Cas12c (C2c3); V-D; V-E; V-F1 (V-U3); V-F2; V-F3; V-G; V-H; V-I; V-J; V-K (V-U5); V-U1; V-U2; V-U4	DNA
	Type VI	Cas 13	VI-A; VI-B1; VI-B2; VI-C; VI-D	RNA

* I-G was I-U (uncharacterized) in 2015.

**Table 3:** Summary of various inherited diseases with germline predispositions to hematologic malignancies with selected references.

Disease Groups	Diseases	Identified Defective Germline Genes	Defective Function or Cellular Pathways	Selected References
Inherited bone marow failure syndromes	Fanconi anemia	At least 22 genes; *FANCA* most common	DNA repair and DNA damage response	Taylor et al., 2019 [127]; Altintas et al., 2023 [128]
	Diamond-Blackfan anemia	*RPS19* most common	Ribosome biogenesis	Wlodarski et al., 2024 [129]; Da Costa et al., 2020 [130]; Liu and Karlsson, 2024 [131]
	Schwachman-Diamond syndrome	*SBDS*, *DNAJC21*, *SRP54*	Ribosome biogenesis	Warren, 2018 [132]; Reilly and Shimamura, 2023 [133]; Kawashima et al., 2023 [134]
	Dyskeratosis congenita and other telomere biology disorders	At least 18 genes; *DKC1* most common	Telomere maintenance	Tummala et al., 2022 [135]; Team Telomere, 2022 [136]
	Severe congenital neutropenia	*ELANE*, *CLPB*, *HAX1*, and *G6PC3*	Myeloid maturation arrest	Warren and Link, 2021 [137]; Donadieu and Bellanné-Chantelot, 2022 [138]
	Congenital amegakaryocytic thrombocytopenia ^1^	*MPL*, *THPO*, *HOXA11*, *MECOM*, *RBM8A*	Megakaryocyic maturation	Balduini, 2023 [139]; Germeshausen and Ballmaier, 2021 [140]
	*ERCC6L2* inherited bone marrow failure	*ERCC6L2*	DNA repair	Bluteau et al., 2018 [141]; Baccelli et al., 2023 [142] Hakkarainen et al., 2023 [143];
Genetic syndromes with predisposition to hematolymphoid cancer	Li–Fraumeni syndrome	*TP53*	Loss of tumor suppressor function	Frebourg et al., 2020 [144]; de Andrade et al., 2021 [145]; Rocca et al., 2022 [146]
	Lynch syndrome	*MLH1*, *MSH2*, *MSH6*, *PMS2*, *EPCAM*	DNA repair	Sandner et al., 2019 [147]
	Constitutional mismatch repair deficiency (CMMRD)	*MLH1*, *MSH2*, *MSH6*, *PMS2*, *EPCAM*	DNA repair	Aronson et al., 2022 [148]; Gallon et al., 2024 [149]
	Bloom syndrome	*BLM*	DNA damage response and repair	Taylor et al., 2019 [127]; Langer et al., 2023 [150]
	Werner syndrome	*WRN*	DNA damage response and repair	Oshima et al., 2017 [151]; Lauper et al., 2013 [152]
	Ataxia telangiectasia	*ATM*	DNA damage response and repair	Taylor et al., 2019 [127]; Petley et al., 2022 [153]; Guijarro et al., 2023 [154]; Riboldi et al., 2024 [155]; Elitzur et al., 2024 [156]
	Nijmegen breakage syndrome	*NBN*	DNA damage response and repair	Taylor et al., 2019 [127]
	DNA ligase 4 deficiency (LIG-4 symdrome)	*LIG4*	DNA damage response and repair	Altmann and Gennery, 2016 [157]; Schober et al., 2019 [158]
	RASopathies	*NFI*, *CBL*, *PTPN11*, *KRAS*, *NRAS*, and other	RAS mitogen-activated protein kinase pathway	Riller and Rieux-Laucat, 2021 [159]; Wintering et al., 2021 [160]; Hecht et al., 2022 [161]
Familial Acute Myeloid Leukemia (AML) and Myelodysplastic Neoplasm (MDN) ^2^	Familial AML with germline mutated *CEBPA*	*CEBPA*	Transcription factor	Pabst et al., 2001 [162]; Tawana et al., 2015 [163]; Tarlock et al., 2021 [164]; Pan et al., 2024 [165]
	Familial platelet disoder with propensity to myeloid malignancies	*RUNX1*	Transcription factor	Brown et al., 2020 [166]; Homan et al., 2021 [167]; Pecci and Balduini, 2021 [168]; Homan et al., 2023 [169]
	*ANKRD26*-related inherited thromboicytopenia	*ANKRD26*	Thrombopoietin-dependent signaling	Bluteau et al., 2014 [170]; Pecci and Balduini, 2021 [168]; Homan et al., 2023 [169]
	*ETV6*-related thrombocytopenia	*ETV6*	Transcription factor	Melazzini et al., 2016 [171]; Pecci and Balduini, 2021 [168]; Homan et al., 2023 [169]
	AML or MDN with germline *DDX41* mutations	*DDX41*	RNA splicing, transcription elongation, and DNA replication	Shinriki et al., 2022 [172]; Makishima et al., 2023 [173]; Huo et al., 2023 [174]; Cheloor-Kovilakam et al., 2023 [175]; Winstone et al., 2024 [176]
	Pediatric MDN or AML with de novo germline or inherited *GATA2* mutations	*GATA2*	Transcription factor	Vincent et al., 2012 [177]; Wlodarski et al., 2016 [178]; Homan et al., 2021 [179]; Santiago et al., 2023 [180]
	Pediatric MDN or AML with de novo germline or inherited *SAMD9* or *SAMD9L* mutations	*SAMD9*; *SAMD9L*		Bluteau et al., 2018 [141]; Sahoo et al., 2021 [181]; Narumi 2022 [182]

^1^ The disease with affected *RBM8A*, termed thrombocytopenia absent radius syndrome, does not progress to thrombocytopenia; platelet counts rise over time [139]; ^2^ The name “myelodysplastic syndromes” was changed to “myelodysplastic neoplasms” by the fifth edition of the World Health Organization diagnostic classification of hematolymphoid tumors in 2022 [183].

## Data Availability

No new data were created or analyzed in this study. Data sharing is not applicable to this article.
